# Characterization of the Rainbow Trout Egg MicroRNA Transcriptome

**DOI:** 10.1371/journal.pone.0039649

**Published:** 2012-06-25

**Authors:** Hao Ma, Mark Hostuttler, Hairong Wei, Caird E. Rexroad, Jianbo Yao

**Affiliations:** 1 Division of Animal and Nutritional Sciences, West Virginia University, Morgantown, West Virginia, United States of America; 2 National Center for Cool and Cold Water Aquaculture, Kearneysville, West Virginia, United States of America; 3 School of Forest Resources and Environmental Science, Michigan Technological University, Houghton, Michigan, United States of America; East Carolina University, United States of America

## Abstract

MicroRNAs (miRNAs) are a class of endogenous small non-coding RNA molecules that regulate post-transcriptional expression of target genes and play important roles in animal development. The objectives of this study were to characterize the egg miRNA transcriptome and identify novel egg-predominant miRNAs in rainbow trout. Small RNAs isolated from mature unfertilized rainbow trout eggs were subjected to deep sequencing using an Illumina Genome Analyzer. The massive sequencing produced 24,621,741 quality reads, among which, 266 known miRNAs were identified and 230 putatively novel miRNAs were predicted. The most abundantly known miRNAs are let-7 and miR-21, accounting for 24.06% and 18.71% of the known miRNAs, respectively. Other known miRNAs which are abundantly present in eggs include miR-24, miR-202, miR-148, miR-30, miR-10, miR-146, miR-25, and miR-143. Real time PCR analysis using cDNAs derived from 10 tissues validated 87 out of 90 selected putative miRNAs and identified three novel miRNAs predominantly expressed in rainbow trout eggs. Each of these novel egg-predominant miRNAs is predicted to target a significant number of genes, most of which are significantly down-regulated in naturally ovulated rainbow trout eggs based on analysis of publicly available microarray data sets. Quantitative real time PCR analysis also demonstrated low expression of a selected number of target genes in eggs relative to liver and muscle tissues. This study represents the first complete survey of miRNAs in fish eggs and provides a starting point for future studies aimed at understanding the roles of miRNAs in controlling egg quality and early embryogenesis in rainbow trout.

## Introduction

The small endogenous non-coding microRNAs (miRNAs) play important roles in a wide range of biological processes, including growth and development [1], [2], [3], proliferation [4], differentiation [5], cancer or other diseases [6], [7], [8], aging and apoptosis [2], [5]. Each miRNA is generated by RNA polymerase II driven transcription of a miRNA gene in the nucleus and cleaved by Drosha to form a pre-miRNA precursor, which is transported to the cytoplasm and further processed by Dicer to form mature functional miRNA [9], [10]. It has been recognized that the actions of miRNA are mainly through degradation of mRNA by perfect or near perfect match of the target RNA, translational suppression by imperfectly complement to the target RNA, and mediated mRNA decay by directing rapid deadenylation of mRNAs [9], [10], [11], [12]. Recent studies have also revealed a less common role of miRNA, that is, miRNAs can stabilize mRNAs and enhance rather than decrease gene expression [13], [14], [15], [16]. Analysis of miRNA targets has revealed that a single miRNA could potentially bind to hundreds of transcripts and one transcript might be regulated by multiple miRNAs [17], [18], [19], [20], [21], [22].

Rainbow trout is one of the most important cold water fish species in the USA due to its importance for food production, sport fisheries, and as a research model [23]. The egg of rainbow trout is especially important because it is one of the determining factors of embryo quality [24], [25], [26], [27]. Production of high quality rainbow trout eggs also has commercial values for egg suppliers. For these reasons, rainbow trout egg quality and production have been extensively studied [25], [28], [29], [30], [31]. Nevertheless, the egg quality can be highly variable [24], [32], partly because it involves multiple developmental phases of oocyte maturation, vitellogenesis, and competent egg ovulation, in which regulation of maternal RNA transcripts and proteins by miRNAs has been implicated [17], [33], [34]. To date, studies of the molecular control of egg quality have focused on the identification and characterization of individual genes as well as the analysis of mRNA stockpile in the eggs [35], [36], [37], [38], [39], [40]. Identification and characterization of egg-expressed miRNAs and discovery of novel egg-predominant miRNAs would be an important step towards understanding of the molecular mechanisms regulating egg quality.

Currently, more than 18,000 miRNA entries have been reported (http://www.mirbase.org/, release 18), which might regulate large proportions of genes in the transcriptome in various cell types. Our initial attempts to clone miRNAs in rainbow trout have resulted in the discovery of a significant number of miRNAs in early stage embryos [41] and pooled somatic tissues [42]. As next generation sequencing technology has been proven to be highly efficient in generating reproducible data, and detecting low-expressed genes [43], it has been used for miRNA discovery in chicken [4], [44], [45], [46], pig [47], cattle [48], and human [49], [50], [51]. The objectives of this study were to provide a global miRNA transcriptomic data of rainbow trout egg, and identify novel or egg-predominant miRNAs that may ultimately be used as molecular markers for egg quality and embryonic development potential.

**Figure 1:**
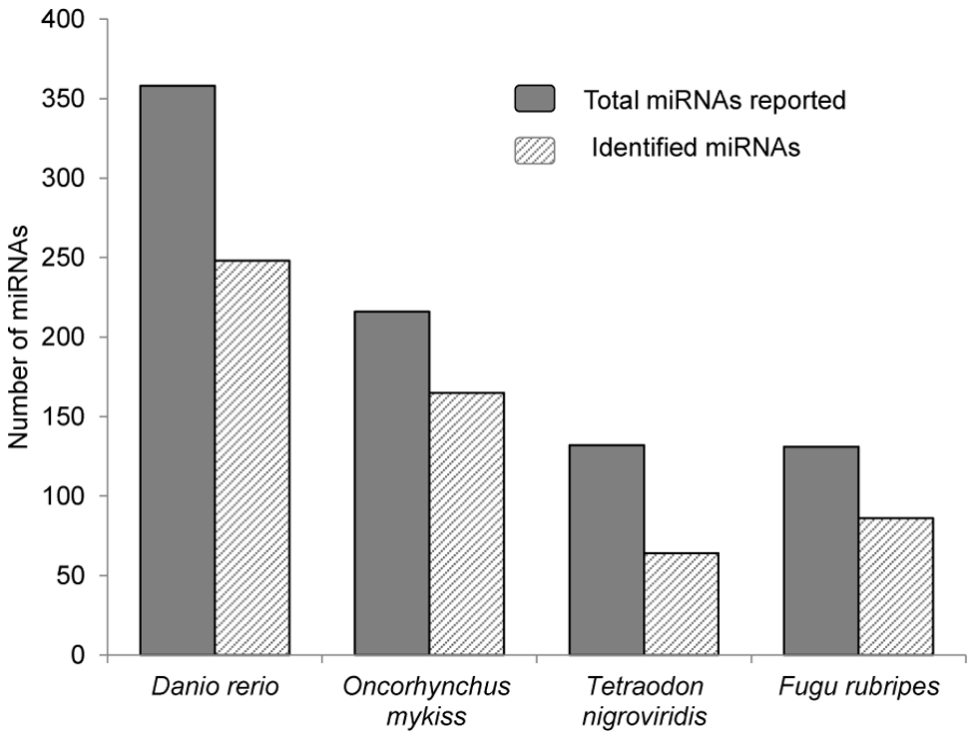
Comparisons of the number of miRNAs (grey bar) of *Danio rerio, Tetraodon nigroviridis, Fugu rubripes* (in miRbase) and *Oncorhynchus mykiss* (published) [**41]**, [42] with the number of known miRNAs (diagonal pattern fill bar) identified in rainbow trout eggs.

**Figure 2:**
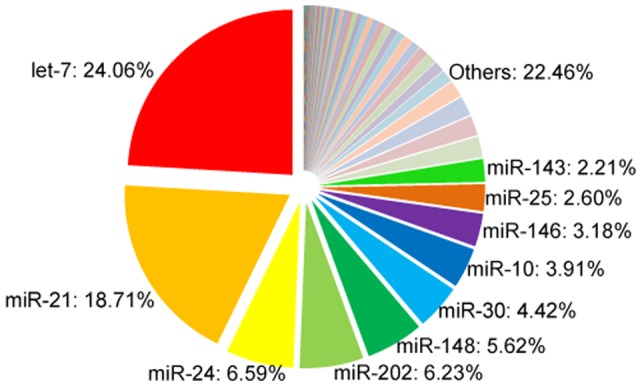
Relative abundance of known miRNAs in rainbow trout eggs.

## Results

### Identification of known miRNAs and their expression profile

A total of 24,621,741 reads were generated from a library constructed with small RNAs isolated from rainbow trout eggs using an Illumina GAIIx. After removing the “impurity” sequences, the remaining alignable reads (11,814,636) were used for identification of known miRNAs and prediction of novel miRNAs. The relative abundance of these mappable reads (15–26 nts) is shown in Figure S1. The majority of them are in the range of 21 to 26 nts, which account for 89.34% of the total mappable reads. The top 4 abundant sizes are 22, 23, 24, and 25, which account for 18.43%, 16.47%, 18.25%, and 15.34% of the total reads, respectively.

Table 1 outlines results of next-generation sequence analysis for miRNAs. Of the 2,358,585 reads that were mapped to miRBase, a total of 8,986 unique sequences were identified which represents 266 miRNA families (Table S1). The identified miRNAs cover a large proportion of reported miRNAs in Pisces, such as zebrafish (*Danio rerio*), spotted green puffer (*Tetraodon nigroviridis*), and Japanese pufferfish (*Fugu rubripes*) (Figure 1). Of the 266 known miRNAs, 152 of them have been reported in our previous studies [41], [42]. The relative abundance of miRNAs varies greatly among the known miRNAs. The top 10 most abundant miRNAs include let-7, miR-21, miR-24, miR-202, miR-148, miR-30, miR-10, miR-146, miR-25 and miR-143, accounting for 77.54% of the 2,358,585 reads mapped to miRBase (Figure 2). As reported in other species, let-7 and miR-21 are the most abundant miRNAs in our data set [51], [52], accounting for 24.06% and 18.71% of the known miRNAs, respectively.

**Table 1:** Number of high-throughput reads and unique sequences generated from a rainbow trout egg small RNA library.

Items	Reads	Unique sequences
Total	24,621,741	4,137,995
Aligned to genome	11,814,636	401,417
Aligned to miRbase	2,358,585	8,986
Unaligned	9,456,051	392,431

### Identification of novel and egg-predominant miRNAs

**Figure 3:**
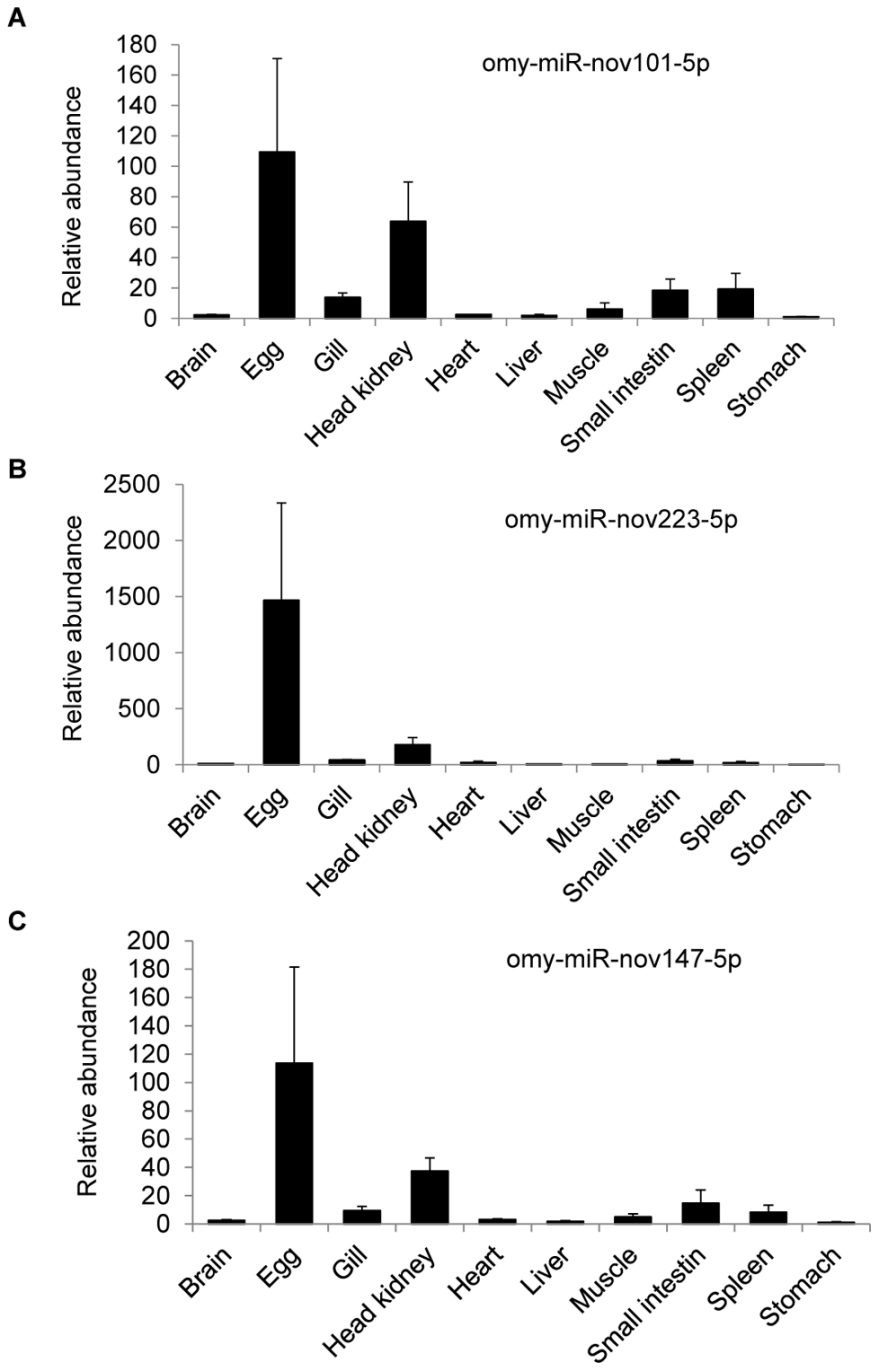
Quantitative real-time PCR analysis showing predominant expression of 3 novel miRNAs in eggs. cDNA samples from 10 tissues including gill, liver, stomach, and head kidney, muscle, egg, heart, small intestine, brain and spleen were used in real time PCR analysis to identify novel miRNAs predominantly expressed in eggs. The means of the normalized miRNA expression values for each sample were calculated and expressed as relative fold changes (n = 3–4, mean ± SEM). Different letters indicate significant difference at P<0.05. (A) omy-miR-nov101-5p. (B) omy-miR-nov223-5p. (C) omy-miR-nov147-5p.

As the genome of rainbow trout has not been sequenced, we used the zebrafish genome sequence to identify potentially novel miRNAs from the miRNAs that were not matched to any sequence in the miRBase. Based on the criteria that the extended sequences (60 nts on both directions) at the aligned genome locations have the propensity of forming hairpin structures, a total of 230 novel miRNAs were predicted (Table S2). To determine if the predicted novel miRNAs contain any egg-predominant miRNAs, we performed real time PCR analysis on 90 randomly selected miRNAs (reads ranging from 3 to 2025) using a panel of cDNA samples from 10 rainbow trout tissues. Of the 90 miRNAs tested, 87 showed specific amplifications as evaluated by melting curve analysis, indicating that these are real miRNAs. Three novel miRNAs (omy-miR-nov101-5p, omy-miR-nov147-5p, and omy-miR-nov223-5p) showed predominant expression in eggs (Figure 3).

### Target prediction of the egg-predominat miRNAs

**Figure 4:**
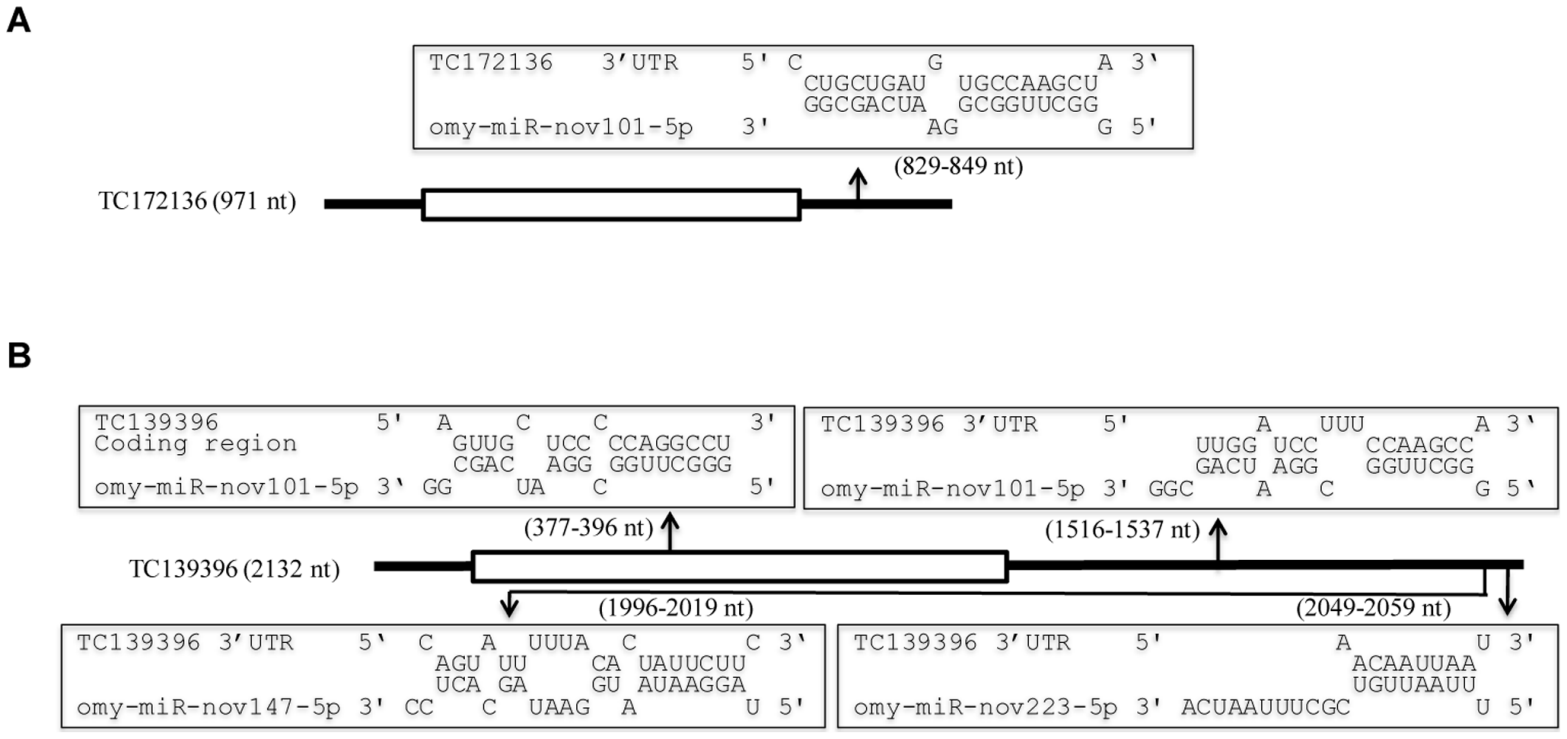
The modes of miRNA targeting for the egg-predominant miRNAs. (A) One miRNA targets a single position in the 3′UTR. (B) Multiple miRNAs target different positions in the same 3′UTR or one miRNA targets both the 3′UTR and the coding region of the same gene. The coding regions are represented by open boxes. Arrows indicate relative locations of the targeting sites.

To provide a better understanding of the roles of these egg-predominant miRNAs, the genes potentially targeted by these miRNAs were predicted using two programs (miRanda and PITA). The prediction was based on the degree of miRNA:target sequence complementarity and the free energy level of RNA-RNA duplexes. As the 3′UTRs are the primary base-pairing region of miRNAs [53], they were screened for binding with the egg-predominant miRNAs first, followed by the coding regions and the 5′UTRs. Only the genes predicted by both programs were considered as potential targets. The numbers of target genes for the egg-predominant miRNAs range from 29 to 198 (Table 2 and Table S4). Some genes are targeted both in the 3′UTRs and the coding regions while no genes are targeted in both the 3′UTRs and 5′UTRs. Seven genes are targeted by both omy-miR-nov101-5p and omy-miR-nov147-5p. In consistence with the known modes of miRNA action, the modes of action of the egg-predominant miRNAs include: 1) one miRNA targets a single position in the 3′UTR (Figure 4A), 2) multiple miRNAs target different positions in the same 3′UTR, and 3) one miRNA targets both the 3′UTR and the coding region of the same gene (Figure 4B).

**Table 2:** Number of target genes of the novel egg-predominant miRNAs predicted by both miRanda and PITA programs.

miRNA	3′UTR	3′UTR+Coding region	3′UTR+5'UTR
omy-miR-nov101-5p	130	26	0
omy-miR-nov223-5p	29	0	0
omy-miR-nov147-5p	193	5	0

### Analysis of target gene expression

**Figure 5:**
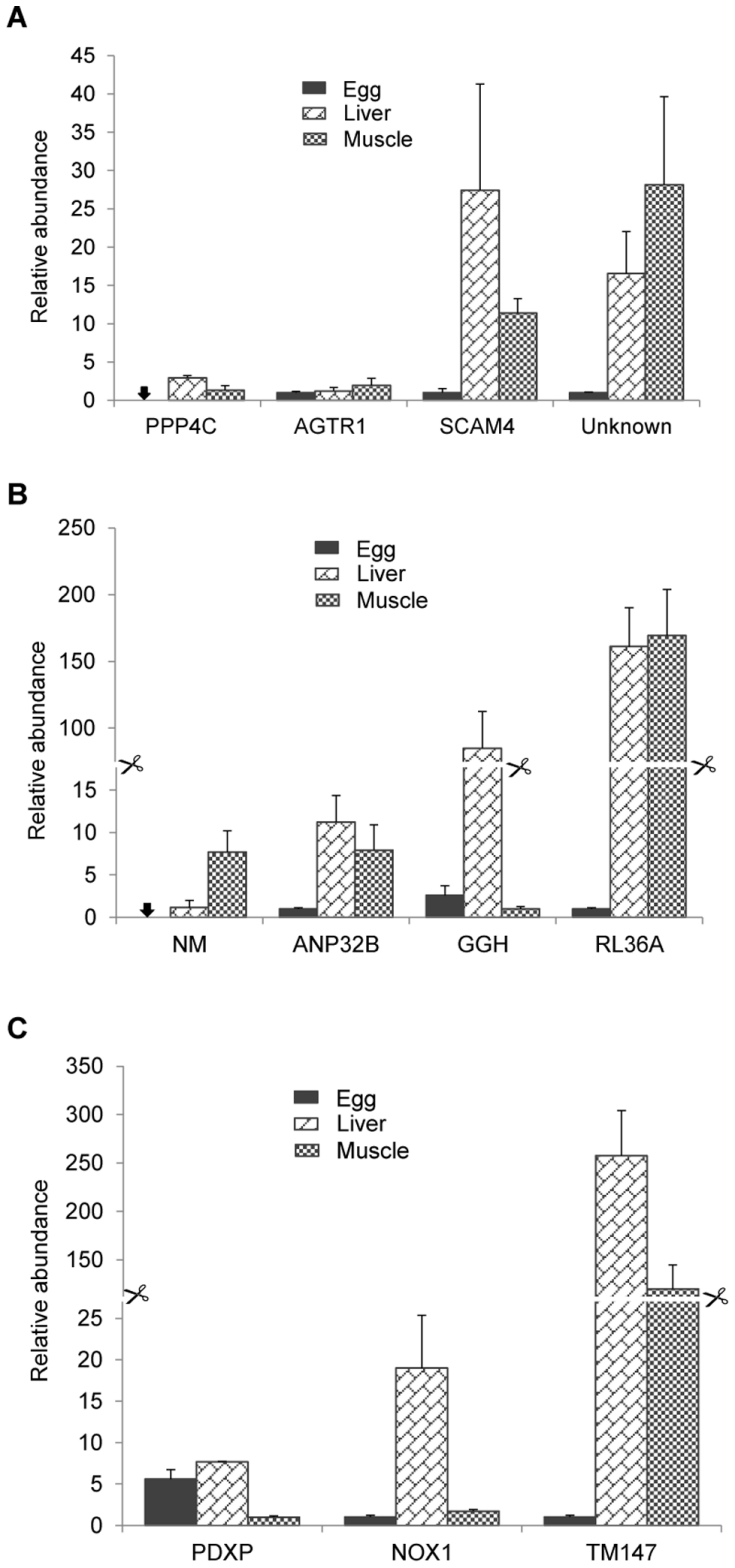
Quantitative real-time PCR analysis of the expression of a selected number of target genes in eggs relative to liver and muscle. cDNA samples from egg, liver, and muscle (n = 3) were used in the analysis. The means of the normalized gene expression values for each sample were calculated and expressed as relative fold changes. Arrows indicate undetectable expression. (A) omy-miR-nov101-5p. (B) omy-miR-nov223-5p. (C) omy-miR-nov147-5p.

In order to shed more light on the functions of the novel egg-predominant miRNAs, microarray gene expression data from naturally ovulated eggs, liver, and muscle samples (GenBank GEO Series: GSE5928, GSE12031, and GSE16577) were used to analyze the expression of the predicted target genes in these tissues. Thirty seven predicted target genes have above 98% sequence similarities to the cDNA entries on the microarrays, with 3 to 18 target genes for each of the 3 egg-predominant miRNAs. A total of 6578 normalized and balanced gene expression values were ranked with small rank value corresponding to low expression level. A Kruskal-Wallis test was performed on the rank values of the target genes to determine the differences in gene expression among egg, liver, and muscle. Although the result did not show statistically significant differences among the tissues, the means of rank values for egg are in general smaller than those for liver and muscle (Table S5). To further evaluate the expression of the target genes in eggs relative to liver and muscle, quantitative real time PCR was used to determine the expression of 3 to 4 selected genes for each miRNA. As shown in Figure 5, the expression of most of the target genes is lower in eggs compared to liver or muscle. This analysis indicates that expression of most of the target genes of the egg-predominant miRNAs is negatively associated with the abundance of the miRNAs.

## Discussion

Fish eggs contain maternal mRNAs and proteins required for early embryonic development after fertilization [26]. The essential roles of miRNAs in regulating maternal mRNAs and supporting early embryonic development have been well documented in zebrafish [17], [54]. Characterization of egg miRNA transcriptome and identification of novel egg-predominant miRNAs is an important step toward the understanding of the molecular mechanisms regulating egg quality. The present study provided a first survey of the miRNAs in rainbow trout eggs. In addition to known miRNAs, putatively novel miRNAs were predicted including three that showed predominant expression in eggs. Further understanding the roles of these novel egg-predominant miRNAs in controlling egg development may ultimately lead to the identification of molecular markers for egg quality.

Under the circumstance where the genome of rainbow trout has not been sequenced, the use of reference genomes from other closely related species is essential for screening miRNAs from rainbow trout deep sequencing data. A previous analysis of BLASTX top-hit species distribution of gene annotations using 447 million rainbow trout transcriptome sequences, indicated that rainbow trout genes had the highest homology to zebrafish (*Danio rerio*) sequences in comparison to other fish species with known genome sequences [42]. Therefore, the zebrafish genome was used as a reference in our analysis to identify miRNAs from our sequencing data. Consistent with the whole genome transcriptome analysis, the identified miRNAs from rainbow trout eggs shared more miRNAs with zebrafish than with two other fish species (*Tetraodon nigroviridis* and *Fugu rubripes*) (Figure 1). Phylogenetic analysis and genetic mapping had suggested a chromosome doubling event in zebrafish [55]. This feature was reflected by known miRNAs identified from our data set (Table S1). For example, for omy-miR-145-5p, omy-miR-199-5p, omy-miR-24-3p, and omy-miR-338-3p, the isoforms of each of these miRNAs appeared at several locations in zebrafish genome. Rainbow trout is in a semi-tetraploid state with extensive recent genome duplication events [23], [56], [57]. It is inferable that there exists a more complex distribution of the miRNAs in the genome of rainbow trout.

Real-time PCR has been proven to be a simple and accurate method to identify and measure the expression levels of miRNAs [58]. Using this approach, we analyzed 90 randomly selected novel miRNAs to determine if they are real miRNAs. Based on melting curve analysis, we validated 87 novel miRNAs that showed specific amplifications. Using a panel of cDNA samples from different tissues, we identified three miRNAs predominantly expressed in eggs. In mammals, genes specifically expressed in oocyte play important roles in oogenesis, ovarian folliculogenesis, fertilization, and early embryonic development [59], [60]. Likewise, as important regulators of gene expression, miRNAs specifically or predominantly expressed in oocyte/egg may control the expression of genes important for the aforementioned processes.

Due to the challenge in experimental validation of miRNA targeted genes, computational prediction remains the only source for a rapid identification of putative miRNA targets [61], [62]. To date, many miRNA target prediction programs have been published. In this study, we chose the widely used algorithms of miRanda and PITA to predict miRNA targets [62], [63], [64]. MiRanda was initially developed for predicting miRNA targets in Drosophila and was later extended to identify miRNA targets in mammals and zebrafish [65]. It relies on the evolutionary relationship between miRNAs and their targets. This tool estimates the energy of physical interaction between sequence matching of miRNA:mRNA pairs in an mRNA, and then calculates a score based on the matches, mismatches, and gaps with a false positive rate of 24% [65]. PITA is a parameter-free model for miRNA-target interaction that computes the difference between the free energy gained from the formation of the miRNA-target duplex and the energetic cost of unpairing the target to make it accessible to the miRNA [66]. This model was proven to be more accurate than many existing algorithms. In addition, it takes into account the secondary structure of the miRNA-target hybrid. A combination of the miRanda and PITA algorithms could result in less reliance on seed matches but greater knowledge of target availability. The use of high score cutoff of miRanda would lead to more reliable prediction of targets in our data set [61], [67]. The mode of miRNA action has been reported mainly through targeting 3′UTR [20], [68]. It has also been reported that some miRNAs coordinately down-regulate multiple targets [69], [70]. In consistence with the above observations, the egg-predominant miRNAs showed diversified modes of targeting, indicating the complicated regulatory networks of these miRNAs.

Using microarray gene expression data from a previous study to identify genes important for egg maturation and ovulation process in rainbow trout [38], we analyzed the expression of the target genes of the egg-predominant miRNAs in eggs relative to liver and muscle. Although the analysis did not show significant differences in the expression levels of the target genes in eggs compared to liver and muscle, real time PCR analysis did show lower expression of many of the target genes in eggs (Figure 5), indicating that the abundance of the target genes in eggs is negatively associated with the abundance of the miRNAs. Recent studies have reported that miRNA activity is suppressed in mouse oocytes and early embryos [71], [72], suggesting that the abundance of miRNAs in eggs may have no effect on the expression of their target genes. Further studies are needed to determine if the egg-predominant miRNAs are responsible for down-regulated expression of the predicted target genes in eggs.

In conclusion, this study represents the first characterization of miRNA transcriptome in fish eggs and provides a starting point for future studies aimed at understanding the roles of miRNAs in controlling egg quality and embryogenesis in rainbow trout.

## Materials and Methods

### Ethics statement

All experiments were conducted under approval of the USDA/ARS National Center for Cool and Cold Water Aquaculture Institutional Animal Care and Use Committee, protocol #50.

### RNA isolation

Mature rainbow trout eggs were collected by gently squeezing the anesthetized females reared under standard condition in the National Center for Cool and Cold Water Aquaculture (Kearneysville, WV). Tissue samples including gill, liver, stomach, head kidney, muscle, heart, small intestine, brain, and spleen were also collected from adult rainbow trout. All samples were frozen in liquid nitrogen and stored in −80°C until extraction of RNA. Total RNA from the eggs and tissue samples was isolated using Trizol reagent (Invitrogen, Carlsbad, CA) according to the manufacturer's instructions. RNA isolated from eggs was further purified by lithium chloride precipitations.

### Sequencing and analysis of egg miRNAs

Sequencing of miRNAs was performed by LC Sciences (Huston, TX). In brief, small RNA fraction of 15–50 nts from egg total RNA was isolated from a 15% Tris-Borate-EDTA-Urea polyacrylamide gel. Following ligations of the small RNAs with SRA 5′ and 3′ adaptors (Illumina, San Diego, CA), the RNAs of 64–99 nts were isolated through gel elution and ethanol precipitation. After reverse transcription, the cDNAs were used for cluster generation on an Illumina's cluster station and then sequenced on an Illumina GAIIx following manufacturer's instructions.

The software package, ACGT101-miR v3.5 (LC Sciences, Houston, TX), was used for analyzing the sequencing data. “Impurity” sequences including sequences with low resolution, copy number less than 3 and length less than 15 nts or larger than 26 nts, adapter sequences, junk sequences (such as > = 80% of A,C, G or T; > = 3 Ns; only A, C, or only G, T), and simple sequence were filtered. In addition, the sequences mapping to the databases of mRNA, RFam and Repbase were also removed. The remaining sequences were used to BLAST against Pisces miRNAs in the miRbase database (release 16) and published rainbow trout miRNAs [41], [42] to identify known miRNAs (mismatch: <2 bases; E-values: <0.005) [73], [74]. The remaining sequences that did not match known miRNAs were mapped to zebrafish (*Danio rerio*) genome to identify potentially novel miRNAs. Novel miRNAs were predicted if the extended sequences at the mapped positions have the propensity of forming hairpin structures.

### Validation and analysis of novel miRNA expression

Ninety predicted novel miRNAs were selected for validation by quantitative real time PCR analysis. To identify novel miRNAs specifically or predominantly expressed in eggs, a panel of 10 samples including eggs was used in the analysis (n = 3–4). Two μg of DNase-treated RNA were converted to cDNA using miScript reverse transcriptase mix (Qiagen, Valencia, CA). The cDNA was then used for real time PCR quantification of miRNAs using miRNA specific primers (Table S3) in combination with the miScript universal primer (Qiagen, Valencia, CA). Rainbow trout β-actin (TC69887) and Histone H2A (TC85036) genes were used as endogenous controls. (primers shown in Table S3). Quantitative real-time PCR was performed on a Bio-Rad CFX96 system. The iQ™ SYBR® Green Supermix (Bio-Rad, Hercules, CA) was used in 20-μl reaction volume containing 100 µM of each primer and 3 µl of diluted (4 times) cDNA. Cycling parameters were 95°C for 3 min followed by 40 cycles of 95°C for 10 sec and 53°C or 60°C for 1 min. Melting curve analyses were programmed following the amplifications. Standard curves for all miRNAs and the endogenous controls were constructed using 10 fold serial dilutions of a pooled cDNA sample. For each sample, the quantity of the specific miRNAs and the reference genes was determined from respective standard curves. The quantity of the specific miRNAs was then divided by the quantity of the reference genes (geometric mean of the 2 reference genes) to obtain a normalized value. Mean differences in expression levels were reported as relative fold changes using the lowest expression value as a calibrator. One way ANOVA was performed to analyze miRNA expression using R. Multiple comparisons were used to determine the differences in miRNA expression among different tissues. Different letters indicate significant differences (P<0.05).

### Identification of miRNA targets via computational analysis

To identify the target genes of the egg-predominant miRNAs, the approximate 3′UTRs of rainbow trout transcripts were first identified. BLASTX was used to identify the homologous genes between zebrafish (*Danio rerio*) protein sequences and gene model sequences of rainbow trout. Zebrafish protein sequences of 36,446 genes were downloaded from Ensembl (http://www.ensembl.org/). After filtering out these genes without sequence, we obtained 32,953 protein sequences. Rainbow trout gene model sequences of 90,018 sequences were obtained from TGI (http://compbio.dfci.harvard.edu/tgi/). The major options for BLASTX included query filter of low complexity sequences, no gaps, and a window size of six, and the cut-off criteria applied to BLASTX output included an E-value of 10 and a match of at least 60 percent to the shorter sequence of either query or subject. If more than one gene was matched, the one with maximal match was regarded as the homolog in rainbow trout. We then fetched 7442 approximate 5′UTRs, 14,788 coding regions and 15,387 3′UTRs for all matched genes. Since 5′UTR typically varies from 60∼90 nts, we cut 60 nts of upstream sequences from the 5′ end matches of protein sequences of zebrafish and cDNA of rainbow trout. The approximate 3′UTRs start from 3′end matches to the end of cDNAs. The cDNA sequences corresponding to the zebrafish protein sequences were treated as the approximate coding regions.

We analyzed 15,387 3′UTRs by two widely used miRNA target prediction algorithms, miRanda (http://www.microrna.org/ microrna/home.do) [65] and PITA (http://genie.weizmann.ac.il/pubs/mir07/mir07_exe.html) [66]. The thresholds of miRanda for candidate target sites were S> = 140 and ΔG<−17 kcal/mol, where S is the sum of single-residue-pair match scores over the alignment trace and ΔG is the free energy of duplex formation from a completely dissociated state which was calculated using the Vienna package [65]. The default parameters were used for PITA [67]. In addition to the 3′UTRs, 5′UTRs and coding sequences were also analyzed.

### Target gene expression analysis

Microarray gene expression data for egg, liver and muscle were obtained from NCBI GenBank database (GEO Series: GSE5928, GSE12031 and GSE16577). The ranked gene expression values were calculated from four egg (GSM137755, GSM137756, GSM137742 and GSM137743), three liver (GSM304071, GSM304072 and GSM304080) and three muscle samples (GSM416734, GSM416735 and GSM416736). A total of 9152 gene expression values were obtained, of which 6578 are balanced values. A Kruskal-Wallis test was performed on the rank values of the target genes to determine the differences in gene expression among egg, liver, and muscle. For real time PCR analysis, three to four target genes for each egg-predominant miRNA were selected with minimum free energy of hybridization between the miRNAs and their target genes ranging from −34.5 to −19.8 Kcal/mol (Table S6). The real time PCR assay was performed as previously described [75] using primers shown in Table S6. Data normalization was performed as described above in the real time PCR analysis of miRNA expression.

## Supporting Information

Figure S1 **Length distribution and abundance of the high-throughput sequences.**
(TIF)

Table S1 **Known miRNAs identified from rainbow trout eggs.**
(XLSX)

Table S2 **Novel miRNAs identified from rainbow trout eggs.**
(XLSX)

Table S3 **Primers used for real-time PCR analysis of miRNA expression.**
(XLSX)

Table S4 **Predicted target genes of the egg-predominant miRNAs.**
(XLSX)

Table S5 **Relative expression levels of genes targeted by egg-predominant miRNAs in egg, liver and muscle.**
(XLSX)

Table S6 **Selected target genes, minimum free energy, and primers used for real time PCR.**
(XLSX)
