# Supplementary figures and images for: Characterization of the Rainbow Trout Egg MicroRNA Transcriptome
Source: PLoS One. 2012 Jun 25;7(6):e39649. doi: 10.1371/journal.pone.0039649 (PMC3382587; doi:10.1371/journal.pone.0039649)

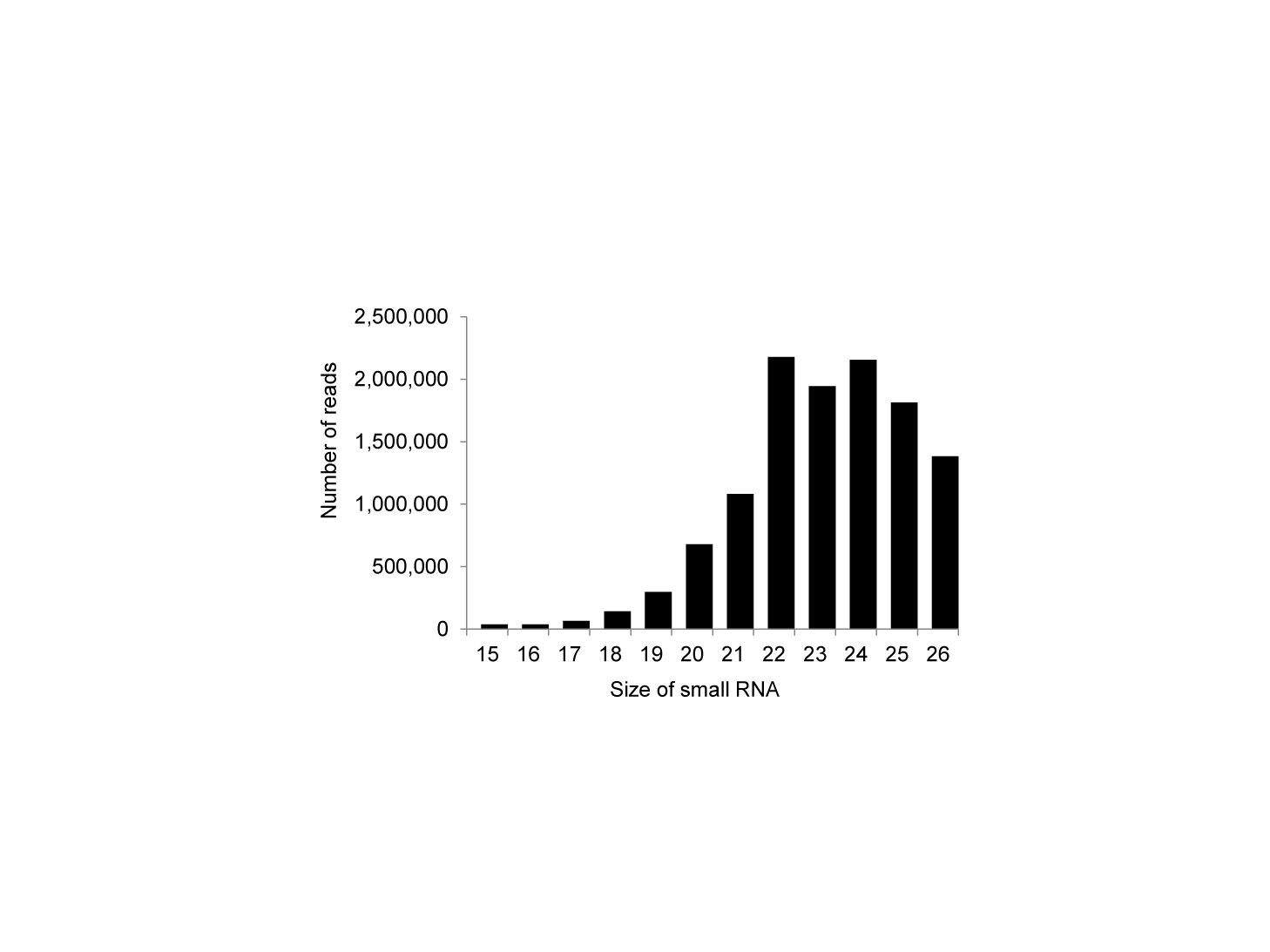

Supplement: Figure S1 — Length distribution and abundance of the high-throughput sequences. (TIF) [file pone.0039649.s001.tif]
